# Profile of Mothers of Children with Fetal Alcohol Spectrum Disorder: A Population-Based Study in Canada

**DOI:** 10.3390/ijerph17217986

**Published:** 2020-10-30

**Authors:** Svetlana Popova, Shannon Lange, Valerie Temple, Vladimir Poznyak, Albert E. Chudley, Larry Burd, Margaret Murray, Jürgen Rehm

**Affiliations:** 1 Centre for Addiction and Mental Health, Institute for Mental Health Policy Research, 33 Ursula Franklin Street, Toronto, ON M5S 2S1, Canada; shannonlange@hotmail.com (S.L.); jtrehm@gmail.com (J.R.); 2 Dalla Lana School of Public Health, University of Toronto, 155 College Street, Toronto, ON M5T 3M7, Canada; 3 Factor-Inwentash Faculty of Social Work, University of Toronto, 246 Bloor Street W, Toronto, ON M5S 1V4, Canada; 4 Institute of Medical Science, University of Toronto, Faculty of Medicine, Medical Sciences, Building, 1 King’s College Circle, Toronto, ON M5S 1A8, Canada; 5 Surrey Place, 2 Surrey Place, Toronto, ON M5S 2C2, Canada; Valerie.Temple@surreyplace.ca; 6 Department of Mental Health and Substance Abuse, World Health Organization, 20 Avenue Appia, CH-1211 Geneva, Switzerland; poznyakv@who.int; 7 Department of Paediatrics and Child Health, University of Manitoba, 840 Sherbrook Street, Winnipeg, MB R3A 1S1, Canada; abchudley@gmail.com; 8 North Dakota Fetal Alcohol Syndrome Center, Pediatrics Department, 1301 N Columbia Rd, Stop 9037 Grand Forks, ND 58202-9037, Canada; larry.burd@und.edu; 9 National Institute on Alcohol Abuse and Alcoholism, National Institutes of Health, Bethesda, MD 20892, USA; pmurray0252@gmail.com; 10 Institute of Clinical Psychology and Psychotherapy & Center of Clinical Epidemiology and Longitudinal Studies, Technische Universität Dresden, Chemnitzer Str. 46, 01187 Dresden, Germany; 11 Department of Psychiatry, University of Toronto, 250 College Street, Toronto, ON M5T 1R8, Canada

**Keywords:** maternal characteristics, pregnancy, prenatal alcohol exposure, fetal alcohol syndrome, fetal alcohol spectrum disorder

## Abstract

Objective: To compare the characteristics of mothers of children with Fetal Alcohol Spectrum Disorder (FASD) with mothers of typically developing control children. Methods: The study utilized a cross-sectional, observational design, using active case ascertainment. Biological mothers were interviewed using a standardized retrospective questionnaire to collect data on demographics, living environment, pregnancy history, nutrition, alcohol and other drug use prior to and following pregnancy recognition. Results: A total of 173 mothers were interviewed. Of these, 19 had a child who was diagnosed with FASD, five had a child who had received a deferred FASD diagnosis, and 37 had children who were selected into the control group as typically developing children. The remaining 112 mothers had children who did not meet diagnostic criteria for FASD. The mothers of children with FASD did not differ significantly from mothers of the control group children with respect to age, ethnicity, marital status, and employment status at the time of pregnancy. However, mothers of children with FASD had lower levels of education (*p* < 0.01) and were more likely to have received financial support (*p* < 0.05) at the time of pregnancy, to have smoked tobacco (*p* < 0.001), and to have used marijuana or hashish (*p* < 0.01) prior to pregnancy recognition, compared with mothers of control children. All mothers of children with FASD reported alcohol consumption prior to pregnancy recognition; however, only 10.5% reported alcohol consumption following pregnancy recognition. None of the mothers interviewed reported any drug use following pregnancy recognition. Conclusions: Population-based preventive interventions, including repeated screening, monitoring, and education regarding the effects of alcohol use, as well as other substances, before and during pregnancy, are needed to eliminate risk for FASD and other negative consequences on child and maternal health.

## 1. Introduction

Globally, alcohol use during pregnancy is one of the most prevalent substances contributing to a pandemic health problem that affects the health of both the mother and her child [1,2,3]. Given that alcohol use during pregnancy is the cause of Fetal Alcohol Spectrum Disorder (FASD) [4], it is of great concern that the prevalence of alcohol use during pregnancy has either remained unchanged or increased in many regions of the world over the last few decades [5]. Recent studies suggest that prenatal exposure to as little as one standard drink per day during pregnancy increases the risk for FASD [6,7,8]. Recent studies using biomarkers in population-based samples suggest that one in 12 women continue to drink until the end of pregnancy, representing a large public health problem [5,9].

Recent global estimates demonstrate that FASD is a common condition—with a global prevalence of 7.7 per 1000 children and youths [10]. In Canada, FASD is currently more common (2–3%) [11]) than autism (1.5%) [12] and is especially prevalent in children in foster care, special education, residential care, and juvenile corrections systems [13].

It now appears likely that a significant fraction of premature maternal mortality, which has recently been increasing, is associated with alcohol use during pregnancy [14,15]. Similarly, premature mortality rates are increased by 2.4% and by 4.5% among the siblings of individuals with FASD when compared to the general population [16,17]. The estimated annual cost of care is $22,810 per child and $24,308 per adult with FASD, exceeding the annual cost of care for those diagnosed with autism, asthma or diabetes [18,19]. Using current diagnostic criteria for FASD, even developed countries with access to diagnostic services respond to only a small proportion of new cases born each year and are frequently unable to extend services to the existing population of older individuals with FASD [20].

Improved information on the profiles of women who drink during pregnancy and subsequently have children with FASD is essential to developing targeted prevention and intervention efforts [19]. This information is also essential to improve identification and treatment of women who continue to drink between pregnancies. Research in this area has demonstrated that using maternal characteristics to risk-stratify populations can improve the efficiency of funding prevention efforts by several hundred times by focusing efforts on the highest risk women [19].

While previous studies on the characteristics of mothers of children with FASD have been published, many of these investigations focused on clinic-based, low-income, high-risk populations [21,22,23]. Results often found a lower level of education in mothers of children with FASD, lower socioeconomic status, and higher levels of alcoholism in their spouses and families [23]. Although information from these groups is important for prevention efforts, alcohol consumption is widely known to occur across a wide range of incomes and demographics, and it is likely that women at risk for drinking during pregnancy, whether they consume alcohol only prior to pregnancy recognition or thereafter, are present in the broader population as well. More recently, May and colleagues reported on the characteristics of women who had a child with FASD in several large population-based samples from across the United States [7,8]. Their results found one of the strongest and most consistent predictors of having a child with FASD was a higher rate of pre-pregnancy drinking (i.e., more than three drinks per day). For other maternal characteristics, however, they found differing results across geographic regions. In some areas, mothers of children with FASD also frequently consumed other drugs such as marijuana, were younger at pregnancy, or were more likely to be unmarried; however, these findings were not consistent across all locations. Based on these results, it would appear that maternal characteristics associated with having a child with FASD may vary depending on factors such as the population studied or the geographic region. This indicates that additional research is needed in this area, and that programs and prevention efforts working to target groups at risk may be most effective if they are based on information specific to a particular population.

In order to improve our understanding of potential risk factors for having a child with FASD and expand the existing literature on this subject, using data from a population-based study of children from Toronto, Canada, the current investigation compared the characteristics of mothers of children with FASD with mothers of the control children.

## 2. Materials and Methods

This study was part of the World Health Organization International Collaborative Research Project on Child Development and Prenatal Risk Factors with a focus on FASD, which aimed to determine the population-based prevalence of FASD among elementary school students, aged 7 to 9 years, who attended public schools in the Greater Toronto Area (GTA) in Ontario, Canada. The GTA is comprised of five regional municipalities and is the most populous metropolitan area in Canada with a total population of 6.42 million, representing 18.3% of Canada’s population [24].

The study utilized a cross-sectional, observational design, using active case ascertainment (i.e., an epidemiological surveillance strategy in which cases are actively sought for examination and diagnosis), along with retrospective collection of prenatal alcohol exposure information from biological mothers. Final diagnostic screening conclusions were made by consensus by a team of experienced multidisciplinary experts during case conferences, using the 2005 Canadian guidelines for FASD diagnosis [25]. A detailed description of the methodology used in this study is available from Popova and colleagues [11].

### 2.1. Maternal Interview

Fetal Alcohol Syndrome (FAS) can be diagnosed without information on prenatal alcohol exposure [25]. However, the information on maternal alcohol history during pregnancy is absolutely required for the diagnosis of partial FAS and Alcohol-related Neurodevelopmental Disorder (ARND) among individuals with behavioral and cognitive difficulties who do not present the specific facial dysmorphologic characteristics of FAS [25]. Therefore, an interview with the biological mother was requested for (1) children who demonstrated deficits (defined as two standard deviations below the mean on a subtest) in a minimum of two domains assessed during the neurodevelopmental assessment and (2) typically developing children (control group). This threshold was set to increase the likelihood that all potential cases were identified, as impairment in a minimum of three domains is necessary for a FASD-specific diagnosis [25]. The 30-min semi-structured interviews were conducted via telephone. During the interview, data were collected on demographics and living environment, pregnancy history, alcohol use (during the past 30 days, lifetime drinking behavior and drinking behavior prior to and following recognition of the pregnancy involving the child in the study), nutrition during pregnancy, and tobacco and other drug use prior to and following pregnancy recognition. The definition of a standard drink was provided to each mother to calibrate the amounts consumed, and drink conversion was done whenever necessary using the standard drink conversion chart. A standard drink is equal to a 341 mL (12 oz) bottle of 5% alcohol beer, cider or cooler; a 142 mL (5 oz) glass of 12% wine; an 85 mL glass of fortified wine (16%–18% alcohol; e.g., sherry, port or vermouth); or a 43 mL (1.5 oz) shot of 40% hard liquor (vodka, rum, rye, whisky or gin).

Interviewers were fully trained on the sensitive nature of the topic of alcohol use during pregnancy and its effects on the family. In addition, the interviewers were blinded as to which mothers of children were selected as controls and which mothers were selected because their children met FASD diagnostic criteria. A minimum of three attempts to contact the biological mother were made.

### 2.2. Prenatal Alcohol Exposure

As per the opinion of the multidisciplinary team of experts in FASD diagnosis and in alignment with the revised Canadian FASD diagnostic guidelines [26], prenatal alcohol exposure was considered to pose a “high risk” if the biological mother reported two or more binge-drinking episodes (four or more standard drinks on a single occasion) or seven or more standard drinks within one week. Prenatal alcohol exposure was considered to pose “some risk” if the biological mother reported alcohol consumption, but at lower than high-risk levels.

### 2.3. Statistical Analysis

Characteristics of the biological mothers of children with FASD were compared with those of the biological mothers of control children. Chi-square tests were used for analysis of categorical variables. For continuous variables, unpaired Student’s *t*-tests for normally distributed data or one-way analysis of variance (ANOVA) were used when comparing two or more groups, respectively. With a statistically significant ANOVA, post-hoc analyses using Tukey’s pairwise comparisons of means with equal variance were performed. Significance was set at α = 0.05. All statistical analyses were performed using Stata 15 (Stata Corporation, TX, USA, 2017) [27].

### 2.4. Ethics

Prospective parent/guardian participants were fully informed about the procedures involved in this study and gave written consent for their child and themselves (in the case of the maternal interview) to participate. Following the interview, mothers received a gift card as a token of appreciation for their time.

The study protocol and all associated materials were reviewed and approved by the Research Ethics Boards at the Centre for Addiction and Mental Health (165/2012) and Health Canada/Public Health Agency of Canada (REB 2012-0052).

## 3. Results

**Figure 1:**
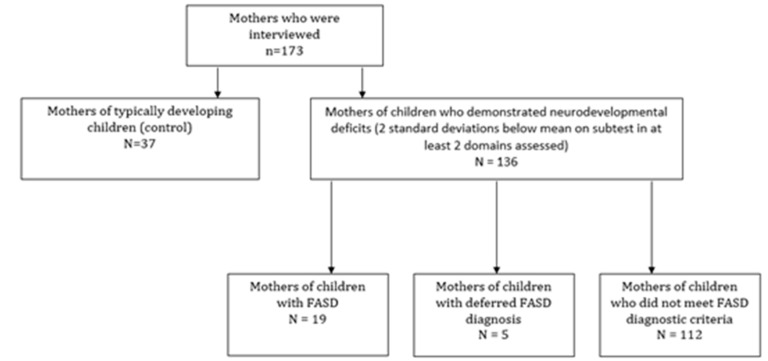
Sampling and recruitment methodology.

A total of 173 biological mothers were interviewed. Of these mothers, 19 had a child who was diagnosed with FASD, five had a child who had received a deferred diagnosis (i.e., prenatal alcohol exposure was identified, but fewer than three central nervous system domains were found to be impaired at the time of assessment), and 37 had a child who did not demonstrate any deviations from the norms and were assigned to the control group as typically developing children. The remaining 112 children of interviewed mothers did not meet diagnostic criteria for FASD. A schematic diagram depicting the sampling and recruitment methodology employed is presented in Figure 1.

### 3.1. Total Sample of Interviewed Mothers

The 173 biological mothers interviewed had a mean age of 40.9 years (*SD* = 5.0; age range: 26–56 years; see Table 1). Almost all mothers were married or living with their partners at the time of their pregnancy (96.0%), were employed in the 12 months leading up to their pregnancy (81.5%), had achieved a post-secondary education (i.e., college diploma, university degree or graduate degree; 83.3%) at the time of their pregnancy, and had planned their pregnancy (72.8%).

In regard to paternal characteristics of the sample (*n* = 173), 93.6% were reportedly employed 12 months leading up to their partner’s pregnancy, and the majority (65.3%) had achieved a post-secondary education at the time of their partner’s pregnancy (Table 2).

Twelve percent of all interviewed mothers received financial support during pregnancy with participating child, and 73% of pregnancies were planned. Mean number of pregnancies was 2.8 (SD 1.3), and 16.2% of children were born prematurely (see Table 3).

Of 173 interviewed mothers, 74.6% reported consuming alcohol (any amount, at any frequency) prior to pregnancy recognition (11.0% reported “high-risk” levels and 63.6% reported “some risk” levels). Only 6.4% of mothers reported alcohol consumption at some-risk levels following pregnancy recognition (Table 4).

Overall, 34.1% (of 173) of mothers had smoked cigarettes prior to pregnancy recognition: 24.3% daily and 9.8% occasionally. Following pregnancy recognition, 86.4% of mothers who smoked before pregnancy recognition quit smoking. Those mothers who continued to smoke during pregnancy (4.6%) did so daily, rather than occasionally. Furthermore, 28.6% of interviewed mothers reported using marijuana or hashish, 4.1% reported using club drugs, 0.6% reported using crack/cocaine and 6.4% reported using hallucinogens prior to pregnancy recognition. No one reported any drug use following pregnancy recognition (see Table 5).

### 3.2. Mothers of Children with FASD Compared with Mothers of Typically Developing Control Children

The mothers of children with FASD did not differ significantly from mothers of control children with respect to age, ethnicity, marital status and employment status, at the time of pregnancy with the child who participated in the study. However, mothers of children with FASD had lower levels of education than mothers of control children at the time of pregnancy (*p* < 0.01). A total of 15.8% of mothers of children with FASD reported receiving financial support (which was at least half of their income) from the child’s grandmother or grandfather (10.5%) and/or from the child’s father (5.3%) during pregnancy. Only 5.4% of mothers of control children reported receiving financial support, and the amount was less than half of their income (*p* < 0.05; see Table 1).

Among mothers of children with FASD, only 63.2% of pregnancies were planned compared with 83.8% among mothers of control children, although the difference was not statistically significant. Compared with mothers of control children, the mean number of pregnancies was higher among mothers of children with FASD (2.7 [*SD* = 1.2] vs. 3.5 [*SD* = 2.3], respectively); more children were born prematurely (10.8% vs. 15.8%, respectively), and more children were born with a birth defect (5.4% vs. 21.1%, respectively). However, none of these differences were statistically significant. Interestingly, mothers of children with FASD had a mean point of pregnancy recognition that was approximately one week earlier than that of mothers of control children (4.4 [*SD* = 1.2] vs. 4.9 [*SD* = 1.8], respectively; however, this difference was not statistically significant; see Table 3).

In terms of paternal characteristics, there were no statistically significant differences in employment status between fathers of children with FASD and fathers of control children. However, a higher proportion of fathers of control children had achieved a post-secondary education at the time of their partner’s pregnancy (86.4%) than fathers of children with FASD (52.7%; see Table 2).

None of the mothers reported having a current drinking problem or ever having sought help for a drinking problem. All mothers of children with FASD reported alcohol consumption prior to pregnancy recognition (high-risk levels: 63.2%, and some-risk levels: 36.8%). Only 10.5% of mothers of children with FASD reported alcohol consumption following pregnancy recognition (some-risk levels only; see Table 4). Significantly more mothers of children with FASD reported ever having smoked tobacco in their lifetime (73.7%), compared with mothers of control children (46.0%; *p* < 0.05; see Table 5).

Moreover, significantly more mothers of children with FASD reported smoking tobacco prior to pregnancy recognition than mothers of control children (68.4% vs. 18.9%, respectively; *p* < 0.001). In addition, the proportion of daily smokers was significantly higher among mothers of children with FASD than mothers of control children (57.9% vs. 8.1%, respectively; *p* < 0.001). Prenatal exposure to both alcohol and cigarette smoking has a multiplicative risk enhancement effect [28].

With respect to substance use prior to pregnancy recognition, there were no significant differences between mothers of children with FASD and mothers of control children, with the exception of marijuana or hashish. The proportion of mothers of children with FASD who used marijuana or hashish was more than double the proportion of mothers of control children (68.4% vs. 27.0%, respectively; *p* < 0.01). Notably, none of the mothers reported any drug use following pregnancy recognition.

## 4. Discussion

This study examined the characteristics of Canadian mothers of a population-based group of children with FASD and compared them with mothers of control children on an array of social, demographic and health attributes. The vast majority of the mothers were from an urban setting, were at least high school-educated, were employed at the time of pregnancy and lived in two-parent families. It was found that mothers of children with FASD did not differ from the mothers of control group children with respect to age, ethnicity, marital status or employment status at the time of their pregnancy. The mothers of children with FASD were more likely to have a lower level of education and to have smoked tobacco and used marijuana or hashish prior to pregnancy recognition.

For all the mothers interviewed, recognition of pregnancy resulted in large decreases in the rates of all substances used. Reported alcohol use for the entire sample dropped from 74.6% to 6.4% (91% reduction) and tobacco smoking from 34.1% to 4.6% (86.6% reduction). A 100% reduction was reported for use of marijuana or hashish (declined from 28.6% prior pregnancy recognition), club drugs from 4.1%, crack/cocaine from 0.6% and hallucinogens from 6.4% to 0%. These results suggest that prevention efforts and public education have likely raised awareness among women in Canada regarding the dangers of substance use during pregnancy and the value of ceasing or reducing use at the time of pregnancy recognition.

Among the mothers of children with FASD, all (100%) reported alcohol consumption before pregnancy recognition and 10.5% reported continuing to drink after discovering they were pregnant. This level of alcohol consumption is in line with recent research in Canada, which has found that between 10% and 15% of women continue to drink while pregnant [29]. Although abstinence during pregnancy would be optimal, these results suggest that rapid widespread risk reduction is possible and underscores the importance of repeated screening, monitoring and education regarding the effects of substance use both before and during pregnancy.

Despite the vast majority of mothers of children with FASD reporting discontinued alcohol use after discovering their pregnancy, these children nevertheless experienced negative effects. This supports the recommendation that there is no safe time to consume alcohol during pregnancy, even in the very early stages of development [26]. It also suggests that women who are planning a pregnancy should avoid alcohol in the preconception period as well as throughout the entire pregnancy [30,31].

Although no women in this study reported using marijuana after discovering their pregnancy, over two-thirds (68%) of mothers of children with FASD reported marijuana use prior to pregnancy recognition. This number is significantly higher than for mothers of the control children (27%) and also substantially higher than for the general population of females across Canada (18%) [32]. Although rates vary widely over time and by population studied, the results reported here are in line with the trend of increasing marijuana usage over the last decade [32,33,34] and suggest this substance may be a growing concern for both maternal and child health. At this time, there is mounting evidence to suggest that prenatal marijuana exposure can result in negative health consequences and impaired functioning [34,35], but these dangers may not be well recognized by the general public. In a series of interviews with pregnant women, Chang and colleagues [36] found that many held the belief that marijuana was “safer” and less harmful to a fetus than other substances because it is more “natural”. In addition, studies have found that information about marijuana use in pregnancy is not as readily available to mothers and is not frequently shared and discussed by healthcare providers [34,37]. This makes increasing public awareness of the possible harms of marijuana use in the perinatal period another priority for public health agencies and primary care providers.

In addition to higher levels of prior marijuana use, mothers of children with FASD also reported significantly higher rates of a history of ever having smoked cigarettes, more daily smoking, and greater concurrent alcohol use and smoking during pregnancy. These results highlight the importance of the previous literature, which found prenatal exposure to both alcohol and tobacco increases risk for a variety of adverse outcomes and highlights the cumulative effects of multiple substance use during pregnancy [28,38]. In addition, as has been reported by others [7,8,35], our results suggest that substance use prior to pregnancy recognition can also increase risk for adverse outcomes and further supports the importance of risk stratification to focus population-level prevention/intervention efforts on those most at risk [19].

Many previous studies of mothers of children with FASD focusing on lower-income groups or clinic-based samples in the United States [22,23,39] have found high levels of addiction, unstable living situations, limited employment and low educational attainment. A few Canadian studies, which used linked administrative Manitobian data, found that mothers who gave birth to children with FASD had higher rates of substance use disorders and psychiatric morbidity [40] and higher rates of suicide [41] than women who gave birth to children without FASD.

A current study contained no women with reported addiction problems; over 80% of the group was employed, and over 95% of had at least a high school education and were living with a partner. Despite this, the mothers of children with FASD were more likely to have received financial support from grandparents or other sources equal to at least half of their income than mothers of control children.

An important limitation of this study is that it relies on retrospective maternal self-reported alcohol consumption, which is subject to social desirability and recall biases [42]. Alcohol use during pregnancy may, therefore, have been underreported and, as a result, some cases of pFAS and ARND may have been missed. This underreporting has also been observed in other studies conducted in the United States and Europe (see, for example, [43,44]).

## 5. Conclusions

The findings in the current study emphasize that FASD is not restricted to disadvantaged groups but rather that it occurs throughout our society regardless of age, ethnicity, marital status, and employment status at the time of pregnancy. These findings contribute to a growing body of evidence, buttressed by strong scientific evidence, demonstrating the presence of a major public health problem. The data suggest and support previous research on the effectiveness and potential cost efficiency of maternal alcohol consumption prevention. Policy makers should not misuse these data for the stigmatization of mothers of children with FASD, but rather they should use it as a tool to develop effective interventions as further research and adequate funding becomes available.

Population-based preventive interventions, including repeated screening, monitoring and education regarding the effects of alcohol use as well as other substances, before and during pregnancy, are needed to eliminate risk for FASD and other negative consequences on child and maternal health in Canada and other countries.

## Figures and Tables

**Table 1:** Maternal demographic characteristics of all interviewed mothers, mothers of children with FASD, and mothers of control children.

Demographics	All Interviewed Mothers (*n* = 173)	Mothers of Children with FASD (*n* = 19)	Mothers of Children Considered Deferred Cases (*n* = 5)	Mothers of Control Children (*n* = 37)	Statistical Test ^a^	*p* Value
Current age (years)—mean (*SD*)	40.9 (5.0)	41.7 (6.0)	42.6 (5.0)	41.4 (4.9)	*t* = 0.188	0.851
Range	26–56	32–49	38–51	30–56		
Ethnicity—*n* (%)					*X* = 7.933	0.160
Caucasian	85 (49.1)	14 (73.7)	2 (40.0)	29 (78.4)		
Aboriginal	1 (0.6)	0 (0.0)	0 (0.0)	0 (0.0)		
African Canadian/Caribbean	11 (6.4)	1 (5.3)	0 (0.0)	0 (0.0)		
Eastern European	12 (6.9)	0 (0.0)	1 (20.0)	3 (8.1)		
Western European	23 (13.3)	4 (21.1)	1 (20.0)	2 (5.4)		
Chinese/Southeast Asian	16 (9.3)	0 (0.0)	1 (20.0)	2 (5.4)		
South Asian	8 (4.6)	0 (0.0)	0 (0.0)	0 (0.0)		
Other	16 (9.3)	0 (0.0)	0 (0.0)	1 (2.7)		
Marital status when pregnant—*n* (%)					*X* = 0.482	0.786
Single	6 (4.5)	1 (5.3)	0 (0.0)	1 (2.7)		
Married, living with husband	137 (79.2)	14 (73.7)	4 (80.0)	30 (81.1)		
Not married, but living with partner	29 (16.8)	4 (21.1)	1 (20.0)	6 (16.2)		
Separated from spouse	1 (0.6)	0 (0.0)	0 (0.0)	0 (0.0)		
Employment status 12 months before pregnancy—*n* (%)					*X* = 0.090	0.764
Employed	141 (81.5)	17 (89.5)	5 (100.0)	34 (91.9)		
Unemployed	32 (18.5)	2 (10.5)	0 (0.0)	3 (8.1)		
Highest level of education completed by pregnancy—*n* (%)					*X* = 15.220	0.004
Less than 9 years	3 (1.7)	1 (5.3)	0 (0.0)	0 (0.0)		
Uncompleted high school diploma	3 (1.7)	0 (0.0)	0 (0.0)	0 (0.0)		
High school diploma	22 (12.7)	5 (26.3)	0 (0.0)	0 (0.0)		
College diploma	56 (32.4)	6 (31.6)	1 (20.0)	9 (24.3)		
University degree	77 (44.5)	6 (31.6)	4 (80.0)	21 (56.8)		
Graduate degree	11 (6.4)	1 (5.3)	0 (0.0)	7 (18.9)		

^a^ Comparing children with FASD with control children; *t*—*t*-score (unpaired Student’s *t*-test); *X*—Chi-square test statistic.

**Table 2:** Paternal demographic characteristics of children with FASD, and control children.

Paternal Characteristics	Based on All Maternal Interviews (*n* =173)	Children with FASD (*n* =19)	Children Considered Deferred Cases (*n* =5)	Control Children (*n* = 37)	Statistical Test ^a^	*p* Value
Employment status 12 months before partner’s pregnancy—*n* (%)					*X* = 0.496	0.780
Employed	162 (93.6)	17 (89.5)	5 (100.0)	35 (94.6)		
Unemployed	5 (2.9)	1 (5.3)	0 (0.0)	1 (2.7)		
Highest level of education completed at time of partner’s pregnancy—*n* (%)						
Less than 9 years	1 (1.2)	0 (0.0)	0 (0.0)	0 (0.0)	*X* = 9.043	0.107
Uncompleted high school diploma	10 (5.8)	3 (15.8)	0 (0.0)	2 (5.4)		
High school diploma	40 (23.1)	4 (21.1)	2 (40.0)	2 (5.4)		
College diploma	46 (26.6)	6 (31.6)	2 (40.0)	12 (32.4)		
University degree	58 (33.5)	3 (15.8)	0 (0.0)	16 (43.2)		
Graduate degree	9 (5.2)	1 (5.3)	1 (20.0)	4 (10.8)		

^a^ Comparing children with FASD with control children; *t*—*t*-score (unpaired Student’s *t*-test); *X*—Chi-square test statistic.

**Table 3:** Pregnancy-related characteristics among all interviewed mothers, mothers of children with FASD and mothers of control children.

Pregnancy-Related Characteristics	All Interviewed Mothers (*n* = 173)	Mothers of Children with FASD (*n* = 19)	Mothers of Children Considered Deferred cases (*n* = 5)	Mothers of Control Children (*n* = 37)	Statistical Test ^a^	*p* Value
Received financial support during pregnancy from relative and/or non-relative	21 (12.1)	3 (15.8)	2 (40.0)	2 (5.4)	*X* = 3.801	0.149
Received financial support was at least half of respondent’s income	14 (8.1)	3 (15.8)	2 (40.0)	0 (0.0)	*X* = 6.187	0.045
Financial supported provided by:						
Child’s grandmother or grandfather	7 (4.1)	2 (10.5)	0 (0.0)	0 (0.0)	*X* = 6.587	0.086
Child’s father	6 (3.5)	1 (5.3)	2 (40.0)	0 (0.0)		
Other relative	3 (1.7)	0 (0.0)	0 (0.0)	1 (2.7)		
Other non-relative	1 (0.6)	0 (0.0)	0 (0.0)	0 (0.0)		
Planned pregnancy—*n* (%)	126 (72.8)	12 (63.2)	5 (100.0)	31 (83.8)	*X* = 5.242	0.155
Number of pregnancies—mean (*SD*)	2.8 (1.3)	3.5 (2.3)	2.8 (1.3)	2.7 (1.2)	*t* = 1.809	0.076
Range	1–11	1–11	1–4	1–7		
Number of live births—mean (*SD*)	2.3 (0.9)	2.5 (1.6)	2.0 (0.7)	2.2 (0.6)	*t* = 0.873	0.387
Range	1–8	1–8	1–3	1–4		
Any children born prematurely (yes)—*n* (%)	28 (16.2)	3 (15.8)	0 (0.0)	4 (10.8)	*X* = 0.285	0.594
Any children with a birth defect (yes)—*n* (%)	20 (11.6)	4 (21.1)	0 (0.0)	2 (5.4)	*X* = 3.213	0.073
Point of pregnancy recognition (weeks)—Mean (*SD*)	4.6 (2.3)	4.1 (1.2)	4.4 (2.6)	4.9 (1.8)	*t* = 1.717	0.092
Range	1–20	1–6	1–8	2–9		

^a^ Comparing children with FASD with control children; *t*—*t*-score (unpaired Student’s *t*-test); *X*—Chi-square test statistic.

**Table 4:** Alcohol use during pregnancy among all interviewed mothers, mothers of children with FASD and mothers of control children.

Alcohol Use	All Interviewed Mothers (*n* = 173)	Mothers of Children with FASD (*n* = 19)	Mothers of Children Considered Deferred Cases (*n* = 5)	Mothers of Control Children (*n* = 37)	Statistical Test ^a^	*p* Value
Lifetime abstainer—*n* (%)	17 (9.8)	0 (0.0)	0 (0.0)	0 (0.0)		
Age of first drink (years)—mean (*SD*)	17.7 (3.0)	16.6 (2.0)	18 (1.4)	17.0 (2.0)	*t* = 0.692	0.492
Age when began to drink regularly (years)—mean (*SD*)	20.7 (5.3)	18.2 (1.7)	20.3 (2.5)	19.4 (3.3)	*t* = 1.381	0.174
Current drinking problem—*n* (%)	0 (0.0)	0 (0.0)	0 (0.0)	0 (0.0)		
Ever sought help for a drinking problem—*n* (%)	0 (0.0)	0 (0.0)	0 (0.0)	0 (0.0)		
Ever felt they should cut down their drinking—*n* (%)	4 (2.3)	0 (0.0)	0 (0.0)	1 (2.7)	*X* = 1.065	0.587
Alcohol use prior to pregnancy recognition—*n* (%)					*X* = 31.605	< 0.001
High risk	19 (11.0)	12 (63.2)	4 (80.0)	0 (0.0)		
Some risk	110 (63.6)	7 (36.8)	1 (20.0)	25 (67.6)		
No risk (no use)	44 (25.4)	0 (0.0)	0 (0.0)	12 (32.4)		
Beverage preference of mothers who used alcohol prior to pregnancy recognition—*n* (%)					*X* = 8.509	0.075
Beer	28 (16.2)	5 (26.3)	1 (20.0)	5 (13.5)		
Wine	76 (43.9)	11 (57.9)	4 (80.0)	15 (40.5)		
Wine coolers or champagne	13 (7.5)	2 (10.5)	0 (0.0)	2 (5.4)		
Liquor/cocktails	13 (7.5)	1 (5.3)	0 (0.0)	4 (10.8)		
Alcohol use following pregnancy recognition—*n* (%)					*X* = 0.496	0.481
High risk	0 (0.0)	0 (0.0)	0 (0.0)	0 (0.0)		
Some risk	11 (6.4)	2 (10.5)	1 (20.0)	2 (5.4)		
No risk (no use)	162 (93.6)	17 (89.5)	4 (80.0)	35 (94.6)		
Beverage preference of mothers who used alcohol following pregnancy recognition—*n* (%)					*X* = 2.469	0.291
Beer	3 (1.7)	0 (0.0)	0 (0.0)	2 (5.4)		
Wine	8 (4.6)	2 (10.5)	1 (20.0)	0 (0.0)		
Wine coolers or champagne	0 (0.0)	0 (0.0)	0 (0.0)	0 (0.0)		
Liquor/cocktails	0 (0.0)	0 (0.0)	0 (0.0)	0 (0.0)		

^a^ Comparing children with FASD with control children; *t*—*t*-score (unpaired Student’s *t*-test); *X*—Chi-square test statistic.

**Table 5:** Tobacco and drug use during pregnancy among all interviewed mothers, mothers of children with FASD and mothers of control children.

Tobacco/Drug Use	All Interviewed Mothers (*n* = 173)	Mothers of Children with FASD (*n* = 19)	Mothers of Children Considered Deferred Cases (*n* = 5)	Mothers of Control Children (*n* = 37)	Statistical Test ^a^	*p* Value
Ever smoked in their lifetime—*n* (%)	81 (47.1)	14 (73.7)	3 (60.0)	17 (46.0)	*X* = 6.525	0.038
Current smoker—*n* (%)						
Daily	21 (12.1)	4 (21.1)	0 (0.0)	2 (5.4)	*X* = 0.278	0.598
Occasionally	6 (3.5)	2 (10.5)	1 (20.0)	2 (5.4)		
Does not smoke	146 (84.4)	13 (68.4)	4 (80.0)	33 (89.2)		
Tobacco use prior to pregnancy recognition—*n* (%)					*X* = 17.233	< 0.001
Daily	42 (24.3)	11 (57.9)	2 (40.0)	3 (8.1)		
Occasionally	17 (9.8)	2 (10.5)	1 (20.0)	4 (10.8)		
Did not smoke	114 (65.9)	6 (31.6)	2 (40.0)	30 (81.1)		
Number of cigarettes smoked per day prior to pregnancy recognition (daily smokers)—mean (*SD*)	6.9 (4.5)	8.1 (6.4)	4.5 (0.7)	4.4 (3.2)	*t* = 1.207	0.248
Range	1–25	1–25	4–5	1–8		
Tobacco use following pregnancy recognition—*n* (%)					*X* = 0.496	0.481
Daily	8 (4.6)	2 (10.5)	0 (0.0)	2 (5.4)		
Occasionally	0 (0.0)	0 (0.0)	0 (0.0)	0 (0.0)		
Did not smoke	165 (95.4)	17 (89.5)	5 (100.0)	35 (94.6)		
Number of cigarettes smoked per day following pregnancy recognition (daily smokers)—mean (*SD*)	4.5 (3.6)	2.5 (0.7)	0 (0.0)	3.0 (1.4)	*t* = 0.447	0.699
Range	1–12	2–3		2–4		
Drug Use						
Drug use during pregnancy (prior to pregnancy recognition)—*n* (%)						
Anabolic steroids	0 (0.0)	0 (0.0)	0 (0.0)	0 (0.0)		
Club drugs (ecstasy, GHB, rohypnol)	7 (4.1)	1 (5.3)	0 (0.0)	2 (5.4)	*X* = 0.001	0.982
Crack/cocaine	1 (0.6)	1 (5.3)	0 (0.0)	0 (0.0)	*X* = 1.983	0.159
Dissociative drugs (PCP, ketamine, salvia, DXM)	0 (0.0)	0 (0.0)	0 (0.0)	0 (0.0)		
Hallucinogens (LSD, mushrooms, peyote)	11 (6.4)	3 (15.8)	0 (0.0)	3 (8.1)	*X* = 0.774	0.379
Heroin or opium	0 (0.0)	0 (0.0)	0 (0.0)	0 (0.0)		
Marijuana or hashish	48 (28.6)	13 (68.4)	2 (40.0)	10 (27.0)	*X* = 8.887	0.003
Methamphetamines/amphetamines	0 (0.0)	0 (0.0)	0 (0.0)	0 (0.0)		

^a^ Comparing children with FASD with control children; *t*—*t*-score (unpaired Student’s *t*-test); *X*—Chi-square test statistic.

## Role of the Funders

The funders had no role in the design of the study; in the collection, analyses, or interpretation of data; in the writing of the manuscript; or in the decision to publish the results.
